# Concordant RNA-protein evidence and human tissue metabolomics prioritize UPP1-associated nucleotide remodeling in prostate cancer

**DOI:** 10.1371/journal.pone.0354354

**Published:** 2026-07-31

**Authors:** Xuancai Chen, Wei Su, Qun Zhou, Yucheng Qi, Juanjuan Xie, Yachun Tang, Xin Tang, Hao Fu

**Affiliations:** 1 Nanhua Hospital, Department of Urology, Hengyang Medical School, University of South China, Hengyang, Hunan, China; 2 Department of Urology, the First Affiliated Hospital of Soochow University, Suzhou, China; Sichuan University, CHINA

## Abstract

Prostate cancer (PCa) is characterized by molecular heterogeneity and metabolic reprogramming, but the relationships among transcriptomic, proteomic, and metabolic signals remain incompletely defined. Here, we present a human-centered, cross-dataset analysis of public RNA, protein, and metabolomics data in PCa. We analyzed RNA and protein profiles from PC3 parental and drug-resistant cells together with an independent human matched tissue metabolomics dataset from Metabolomics Workbench (ST000784). RNA-protein overlap analysis identified seven genes that were significant at both layers, including five concordantly upregulated genes: *UPP1*, *IGF2R*, *FLNC*, *DSP*, and *PLEC*. Among these, *UPP1* provided the most direct metabolic interpretation because it encodes uridine phosphorylase 1, an enzyme linked to pyrimidine salvage. Independent ST000784 matched prostate tissue metabolomics supported broader nucleotide metabolism remodeling, including significant changes in N-carbamoyl-L-aspartate, guanosine monophosphate, and adenosine 3,5-cyclic monophosphate. In contrast, uracil was not significantly altered and uridine 5’-monophosphate showed only a trend after multiple-testing correction. Repeated stratified cross-validation showed that UPP1-containing candidate gene sets achieved high within-layer AUC values in RNA and protein data, while nested statistical benchmark models also performed strongly. These results prioritize a UPP1-associated nucleotide remodeling hypothesis in PCa, but do not establish causal regulation of metabolite abundance or clinical diagnostic utility. Future matched multi-omics and perturbation experiments are required.

## Introduction

Prostate cancer (PCa) is one of the most common malignancies among men worldwide and remains a major cause of cancer-related mortality [1]. The disease is clinically heterogeneous, with prognosis and treatment decisions influenced by pathological grade, tumor stage, androgen receptor signaling state, and molecular subtype [2–7]. Some tumors follow an indolent course, whereas others progress to metastatic or castration-resistant disease. Improved understanding of the molecular and metabolic programs associated with PCa progression is therefore important for biomarker development and for identifying candidate biological vulnerabilities.

Metabolic reprogramming is a recognized hallmark of PCa biology [8–11]. Compared with many other cancers, prostate tumors show prominent changes in lipid synthesis, cholesterol handling, amino acid metabolism, and mitochondrial function [12–19]. These metabolic shifts are shaped by androgen receptor signaling, oncogenic transcriptional programs, tumor microenvironmental pressures, and therapeutic resistance. In addition to lipid metabolism, nucleotide metabolism is increasingly relevant because proliferating and therapy-adapted cancer cells require sustained nucleotide pools for DNA replication, RNA synthesis, repair, and stress adaptation.

High-throughput omics technologies now allow the molecular state of PCa to be profiled at multiple levels, including genomics, transcriptomics, proteomics, phosphoproteomics, and metabolomics [20–29]. Multi-omics integration can help prioritize candidates that are supported by more than one biological layer [30–39]. However, true sample-matched multi-omics integration requires measurements from the same specimens or matched biological replicates. When public datasets are derived from different cohorts, models, or sample types, the appropriate interpretation is more conservative: cross-dataset evidence can generate and prioritize biological hypotheses, but cannot by itself establish causal molecular mechanisms.

In this study, we used independent public RNA, protein, and human tissue metabolomics datasets to ask whether concordant RNA-protein evidence can prioritize a metabolically interpretable candidate module, and whether independent human tissue metabolomics supports nucleotide metabolism remodeling in PCa. The analysis was designed as cross-dataset candidate prioritization rather than sample-matched multi-omics integration. Classifier analyses were evaluated using repeated cross-validation with nested benchmark feature selection and are interpreted as within-layer exploratory discrimination rather than as clinical-grade classifiers.

## Materials and methods

### Data acquisition and analysis units

This study reanalyzed publicly available RNA, protein, and metabolomics data. RNA and protein profiles were obtained from the PC3 parental and drug-resistant cell dataset reported by Woo et al. [40]. The proteomics data are available through ProteomeXchange/PRIDE under accession PXD059079 [41]. In the processed matrices used for this reanalysis, the RNA layer contained 7,511 genes across 27 profiles, and the protein layer contained 1,487 proteins across 144 profiles. These RNA and protein profiles were analyzed as the available profile-level units in the processed data. The present analysis started from the processed feature-by-profile matrices provided with the source study; raw-read alignment, UMI-level filtering, and cell-level quality control were not repeated here.

Human tissue metabolomics was obtained from Metabolomics Workbench study ST000784 (https://doi.org/10.21228/M80D5X) [42]. This dataset contains matched prostate cancer and adjacent benign tissue samples. After parsing the local sample metadata, 46 matched pairs were available for paired analysis, including 33 African American (AA) and 13 European American (EA) matched pairs, for a total of 92 tissue samples. The metabolomics matrix contained 188 metabolites with complete paired cancer/benign measurements used in the analysis.

The RNA/protein and ST000784 metabolomics datasets were not measured from the same samples. Therefore, this study treats them as independent datasets and uses metabolomics as human tissue pathway-level context rather than as sample-matched validation of RNA/protein changes. No cross-dataset batch correction or feature-level data fusion was performed because the molecular layers were not combined into a single sample-matched matrix.

### Ethics statement

This study used only publicly available, de-identified datasets; therefore, ethical approval and consent were not required.

### RNA and protein differential analysis

RNA and protein differential analyses were performed independently using the processed feature matrices. For the RNA layer, genes were compared between PC3 parental and drug-resistant profiles. For the protein layer, protein abundances were compared between the corresponding parental and drug-resistant profiles. Differential results were summarized by log2 fold change and p-value, followed by Benjamini-Hochberg false discovery rate (BH FDR) correction. Features with BH FDR < 0.05 were considered significant. This analysis identified 57 significant RNA genes and 1,069 significant proteins.

### RNA-protein overlap and concordance analysis

Gene symbols were used to match RNA and protein features. RNA-protein overlap genes were defined as genes significant at both layers (RNA BH FDR < 0.05 and protein BH FDR < 0.05). Concordant genes were those with RNA and protein log2 fold changes in the same direction. Discordant genes were significant at both layers but had opposite RNA and protein log2 fold-change directions.

### Human tissue metabolomics reanalysis

ST000784 cancer and benign tissue samples were analyzed as matched pairs by subject. For each metabolite, cancer-versus-benign differences were tested using both paired t-tests and Wilcoxon signed-rank tests. BH FDR correction was applied across all 188 measured metabolites for each test family. Nucleotide- and pyrimidine-related metabolites were selected a priori for focused reporting based on biochemical relevance to nucleotide metabolism and UPP1-adjacent pathways. Uracil and uridine 5’-monophosphate (UMP) were retained in the figure even when not FDR-significant to transparently evaluate direct UPP1-proximal metabolites.

### STRING protein-protein interaction context

Protein-protein interaction (PPI) context was visualized using STRING-derived interaction data [43]. The PPI panel was used only to show known associations among overlap genes and neighboring protein nodes. It was not interpreted as condition-specific PPI rewiring or as causal evidence.

### Classifier construction and evaluation

Classifier analyses were restricted to the RNA and protein layers separately. No classifier combined RNA, protein, and metabolomics features because the metabolomics samples were independent from the RNA/protein profiles. Logistic regression models were implemented in Python using scikit-learn [44]. Each pipeline included median imputation, standard scaling, and logistic regression. Candidate feature sets included *UPP1*, *IGF2R*, *UPP1* + *IGF2R*, the concordant-5 set (*UPP1*, *IGF2R*, *FLNC*, *DSP*, *PLEC*), and the overlap-7 set (*UPP1*, *IGF2R*, *FLNC*, *DSP*, *PLEC*, *PCNA*, *ANP32A*).

Model performance was evaluated with repeated stratified 5-fold cross-validation with 50 repeats, producing 250 fold-level AUC estimates for each feature set and molecular layer. Benchmark models used nested top-k feature selection (k = 5, 10, or 20), where features were selected only within each training fold using training-set differential statistics before model fitting. AUC distributions, means, standard deviations, and approximate 95% confidence intervals were summarized. Because independent biological replicate identifiers were not available in the processed RNA/protein matrices used here, cross-validation was performed at the processed-profile level and is reported only as exploratory within-dataset discrimination. The confidence intervals describe resampling variability and do not substitute for independent cohort validation.

## Results

### Human-centered cross-dataset study design

**Fig 1:**
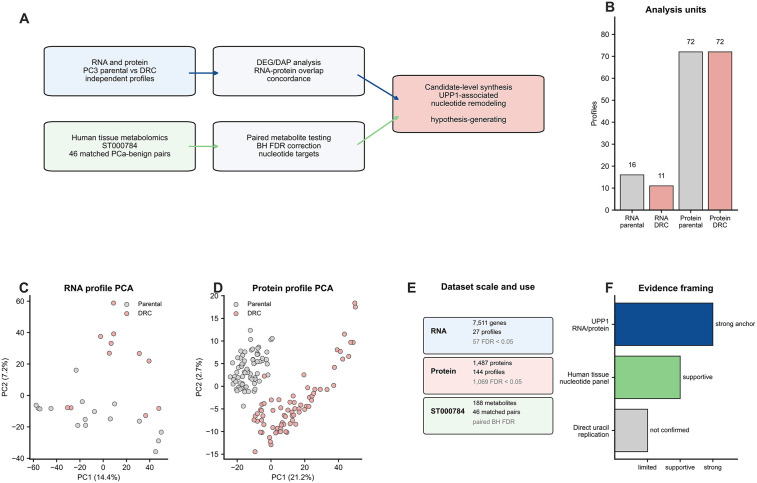
Human-centered cross-dataset study design. (A) Analysis workflow linking RNA/protein profile analysis with independent human matched tissue metabolomics. (B) Analysis-unit summary for RNA and protein profiles. (C-D) PCA visualization of RNA and protein profile structure. (E) Dataset scale and use. (F) Evidence framing. RNA/protein and ST000784 metabolomics data are independent datasets and were not interpreted as sample-matched multi-omics.

This analysis uses a human-centered cross-dataset design rather than a direct sample-matched three-layer integration (Fig 1). RNA and protein data were analyzed from PC3 parental and drug-resistant cell profiles, whereas metabolomics evidence was obtained from ST000784 human matched prostate tissue samples. The RNA matrix included 7,511 genes across 27 profiles, and the protein matrix included 1,487 proteins across 144 profiles. ST000784 included 46 matched cancer-benign pairs (92 tissues), with 33 AA and 13 EA matched pairs and 188 measured metabolites. Because the RNA/protein and metabolomics data were not measured in the same samples, downstream synthesis was interpreted as candidate-level and pathway-level evidence.

### RNA-protein overlap identifies seven shared significant genes

**Fig 2:**
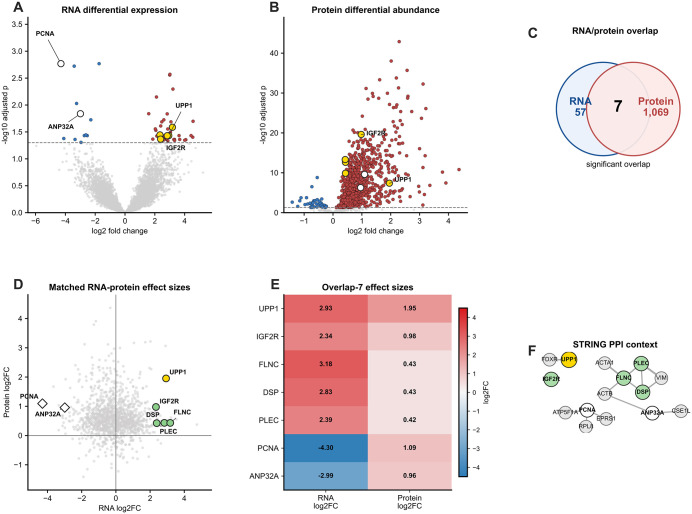
RNA-protein overlap identifies UPP1 within a concordant candidate module. (A) RNA differential expression landscape. (B) Protein differential abundance landscape. (C) Significant RNA/protein overlap, showing 57 significant RNA genes, 1,069 significant proteins, and seven shared genes. (D) Matched RNA-protein effect sizes for common genes, with overlap candidates highlighted. (E) Heatmap of RNA and protein log2 fold changes for overlap-7 genes. (F) STRING known interaction context for overlap candidates and neighboring proteins.

We first compared differential results across the RNA and protein layers. The RNA layer contained 57 significant genes, and the protein layer contained 1,069 significant proteins. Intersecting these two sets identified seven genes significant at both levels (Fig 2A-C; Table 1). Five genes were concordantly upregulated at both RNA and protein levels: *UPP1*, *IGF2R*, *FLNC*, *DSP*, and *PLEC*. Two genes, *PCNA* and *ANP32A*, were significant at both layers but directionally discordant, with lower RNA abundance and higher protein abundance in the drug-resistant profiles (Fig 2D-E).

**Table 1:** RNA-protein overlap genes.

Gene	RNA log2FC	RNA BH FDR	Protein log2FC	Protein BH FDR	Concordant
UPP1	2.934	0.0370	1.953	4.04e-08	Yes
IGF2R	2.343	0.0359	0.977	2.42e-20	Yes
FLNC	3.179	0.0259	0.432	1.28e-10	Yes
DSP	2.832	0.0372	0.433	2.36e-13	Yes
PLEC	2.387	0.0430	0.423	4.76e-14	Yes
PCNA	−4.296	0.00170	1.093	2.56e-10	No
ANP32A	−2.990	0.0145	0.957	5.22e-07	No

Among the concordant genes, *UPP1* was the most directly metabolically interpretable candidate because it encodes uridine phosphorylase 1, an enzyme involved in pyrimidine salvage. *IGF2R* was retained as a concordant co-marker, while *FLNC*, *DSP*, and *PLEC* were interpreted as part of a broader concordant candidate module related to cellular state and structural remodeling. A compact STRING PPI panel provided known interaction context for the overlap candidates and neighboring proteins (Fig 2F).

### UPP1 and IGF2R are concordantly elevated within a candidate module

**Fig 3:**
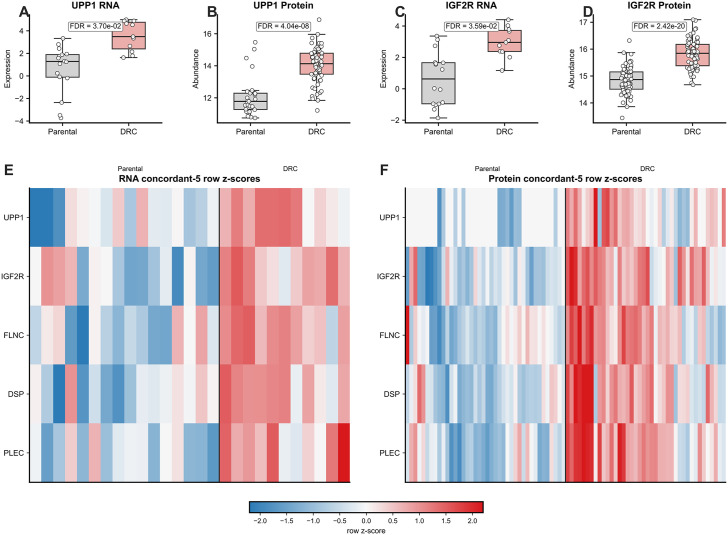
UPP1 and IGF2R are concordantly elevated within the candidate module. (A-B) UPP1 RNA expression and protein abundance. (C-D) IGF2R RNA expression and protein abundance. (E-F) RNA and protein row z-score heatmaps for the concordant-5 module (*UPP1*, *IGF2R*, *FLNC*, *DSP*, and *PLEC*)..

We next examined the two most prominent concordant candidates, *UPP1* and *IGF2R*, at both RNA and protein levels (Fig 3A-D). *UPP1* RNA expression was increased in drug-resistant profiles (RNA BH FDR = 0.037), and UPP1 protein abundance was also increased (protein BH FDR = 4.04e-08). *IGF2R* showed a similar concordant pattern, with increased RNA expression (RNA BH FDR = 0.0359) and increased protein abundance (protein BH FDR = 2.42e-20). Thus, both UPP1 and IGF2R were supported at the RNA and protein levels.

Heatmaps of the concordant-5 module showed that these candidates collectively distinguish parental and drug-resistant profiles at both molecular layers (Fig 3E-F). These results support a concordant candidate module centered on UPP1 and IGF2R, but they do not establish a direct mechanistic relationship among all module members.

### Human matched tissue metabolomics supports nucleotide remodeling but not direct uracil confirmation

**Fig 4:**
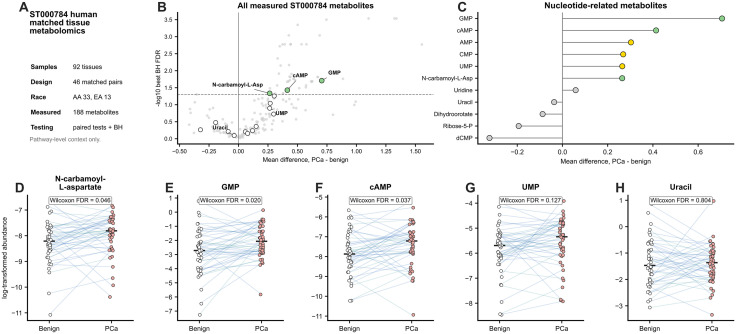
Human matched tissue metabolomics supports nucleotide remodeling without direct uracil confirmation. (A) ST000784 matched tissue metabolomics design. (B) Overview of all measured ST000784 metabolites by mean cancer-minus-benign difference and BH FDR. (C) Nucleotide-related metabolite effect-size summary. (D-H) Matched-pair plots for N-carbamoyl-L-aspartate, GMP, cAMP, UMP, and uracil. UMP is shown as trend-only, and uracil is non-significant. ST000784 metabolomics samples were not matched to the RNA/protein profiles.

We then reanalyzed ST000784 human matched prostate tissue metabolomics (Fig 4). Across 46 matched cancer-benign pairs, paired testing identified several nucleotide-related signals. N-carbamoyl-L-aspartate, a pyrimidine biosynthesis-related intermediate, was higher in cancer tissue by Wilcoxon testing after BH correction (Wilcoxon FDR = 0.046). Guanosine monophosphate (GMP) was also increased in cancer tissue (Wilcoxon FDR = 0.020), and adenosine 3,5-cyclic monophosphate (cAMP) showed a significant matched-pair signal (Wilcoxon FDR = 0.037).

In contrast, direct UPP1-proximal metabolites were not consistently confirmed. Uridine 5’-monophosphate (UMP) showed an upward trend but did not remain significant after BH correction (Wilcoxon FDR = 0.127). Uracil was not significantly altered in the matched human tissue data (Wilcoxon FDR = 0.804). Therefore, the human tissue metabolomics result supports broader nucleotide metabolism remodeling in human PCa tissue, but it does not support the stronger claim that elevated UPP1 directly drives uracil accumulation.

### Candidate gene sets show within-layer discrimination with strong benchmark performance

**Fig 5:**
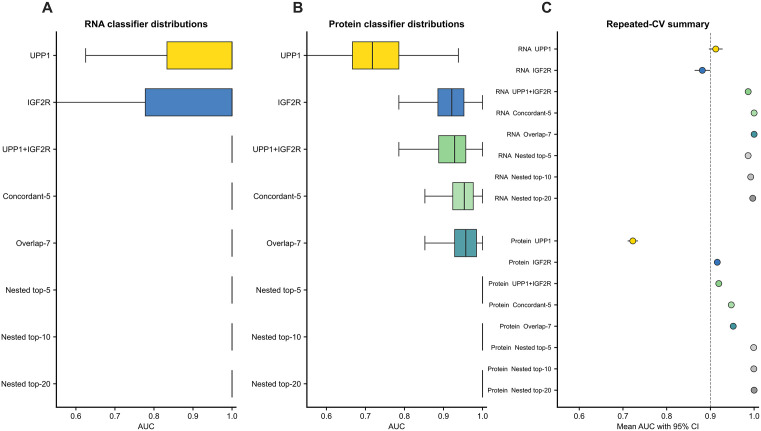
Candidate gene sets show within-layer discrimination, with strong benchmark performance. (A-B) Repeated cross-validation AUC distributions for RNA and protein classifiers. (C) Mean AUC with 95% confidence intervals. Models were evaluated within each molecular layer only. Candidate gene sets were discriminative within the available profiles, while nested statistical benchmark feature sets were also strong.

Finally, we evaluated whether the UPP1-associated candidate sets had discriminative value within the RNA and protein layers (Fig 5). To avoid reliance on a single data split, the analysis used repeated stratified 5-fold cross-validation with 50 repeats and evaluated RNA and protein models separately. In the RNA layer, *UPP1* alone achieved a mean AUC of 0.912, *UPP1* + *IGF2R* achieved a mean AUC of 0.986, and both the concordant-5 and overlap-7 sets achieved mean AUC values of 1.000. In the protein layer, UPP1 alone showed weaker discrimination (mean AUC = 0.723), while *UPP1* + *IGF2R*, concordant-5, and overlap-7 achieved mean AUC values of 0.919, 0.948, and 0.952, respectively.

Nested benchmark models also performed strongly. In particular, the protein nested top-20 benchmark achieved a mean AUC of 1.000. AUC means, standard deviations, and approximate 95% confidence intervals are summarized in Table 2. These results indicate that candidate gene sets can discriminate the available parental and drug-resistant profiles within each molecular layer, but they do not demonstrate better performance than statistical benchmarks or establish clinical diagnostic performance.

**Table 2:** Repeated cross-validation AUC summary.

Layer	Feature set	Mean AUC	SD	Approximate 95% CI
RNA	UPP1	0.912	0.127	0.896-0.928
RNA	IGF2R	0.882	0.142	0.864-0.899
RNA	UPP1 + IGF2R	0.986	0.046	0.981-0.992
RNA	Concordant-5	1.000	0.000	1.000-1.000
RNA	Overlap-7	1.000	0.000	1.000-1.000
RNA	Nested top-5	0.986	0.060	0.979-0.994
RNA	Nested top-10	0.992	0.036	0.988-0.997
RNA	Nested top-20	0.997	0.025	0.994-1.000
Protein	UPP1	0.723	0.096	0.711-0.734
Protein	IGF2R	0.916	0.047	0.910-0.922
Protein	UPP1 + IGF2R	0.919	0.049	0.913-0.925
Protein	Concordant-5	0.948	0.036	0.943-0.952
Protein	Overlap-7	0.952	0.037	0.948-0.957
Protein	Nested top-5	0.999	0.003	0.999-0.999
Protein	Nested top-10	0.999	0.003	0.999-1.000
Protein	Nested top-20	1.000	0.000	1.000-1.000

## Discussion

This study prioritizes a UPP1-associated nucleotide remodeling hypothesis in PCa using a conservative cross-dataset analysis. The RNA-protein overlap analysis identified seven shared significant genes, of which five were concordantly upregulated. UPP1 emerged as the most metabolically interpretable candidate because it encodes uridine phosphorylase 1, which connects directly to pyrimidine salvage biology. However, the available evidence does not prove that UPP1 causally drives metabolite abundance.

UPP1 has been studied in several cancer types, though its role in prostate cancer remains underexplored relative to other metabolic enzymes. In lung adenocarcinoma, UPP1 overexpression was shown to promote tumor progression through the induction of an immunosuppressive microenvironment, in part via TGF-β1 release and PD-L1 upregulation through the PI3K/AKT/mTOR pathway [46]. UPP1 has also been reported to modulate 5-fluorouracil sensitivity in colorectal and pancreatic cancer models through its role in uridine homeostasis and pyrimidine metabolism [47,48]. In PCa specifically, UPP1 has been identified in transcriptomic signatures of aggressive disease [6,7], but dedicated functional studies of UPP1 in prostate cancer models are currently lacking. IGF2R (also known as the cation-independent mannose 6-phosphate receptor, CI-MPR) is a multifunctional receptor with diverse roles in lysosomal enzyme trafficking, TGF-β activation, and insulin-like growth factor 2 clearance [49,50]. In cancer, IGF2R has been reported to exhibit both tumor-suppressive and tumor-promoting functions depending on tissue context: loss of heterozygosity at the IGF2R locus has been described in breast and liver cancers, consistent with a potential tumor-suppressive role, while overexpression has been observed in other settings [50]. The present study provides concordant RNA-protein evidence that both UPP1 and IGF2R are upregulated in drug-resistant PCa cells, extending prior single-layer observations and motivating further functional investigation in PCa.

The human tissue metabolomics analysis is central to the study interpretation. ST000784 supports broader nucleotide metabolism remodeling through significant matched-pair changes in N-carbamoyl-L-aspartate, GMP, and cAMP. At the same time, the human tissue data do not confirm significant uracil elevation, and UMP remains trend-only after multiple-testing correction. This distinction is important: the data support a nucleotide remodeling context around UPP1, but not a direct UPP1-product causal axis.

The metabolomic findings fit within a broader literature on PCa metabolic reprogramming, but the specific metabolic features deserve careful delineation. Lipid metabolism is arguably the most extensively characterized metabolic hallmark in PCa: fatty acid synthase (FASN) is overexpressed in a substantial fraction of prostate tumors and has been proposed as a metabolic oncogene [13]; androgen signaling directly regulates lipogenic enzyme expression [17,18]; and cholesterol accumulation has been observed in prostate cancer tissue [15]. Amino acid metabolism, particularly sarcosine and polyamine pathways, has also been interrogated in PCa metabolomics studies [9,28]. In comparison, nucleotide and pyrimidine metabolism has received less focused attention in the PCa literature, despite its direct relevance to replication, transcription, and DNA repair demand in proliferating and therapy-adapted cells. The present findings—N-carbamoyl-L-aspartate (a pyrimidine de novo synthesis intermediate), GMP, and cAMP alterations in human prostate cancer tissue—suggest that nucleotide pathway remodeling may represent a metabolically coherent and relatively underexplored dimension of PCa metabolic adaptation. These results do not contradict the established centrality of lipid metabolism in PCa; rather, they suggest that nucleotide metabolism may be an additional, complementary layer of metabolic reprogramming.

The concordant elevation of IGF2R was also observed at RNA and protein levels. We interpret IGF2R in this analysis as a concordant co-marker—a gene with consistent multi-layer evidence of altered abundance in drug-resistant cells—rather than as a direct metabolic enzyme. This interpretation is based on the fact that IGF2R’s primary annotated functions involve receptor-mediated trafficking and ligand clearance, not enzymatic catalysis of small-molecule metabolic intermediates [49]. However, we emphasize that alternative interpretations are equally plausible given the absence of functional perturbation data: IGF2R upregulation could reflect a compensatory anti-proliferative response, a bystander effect of broader transcriptional reprogramming in drug-resistant cells, or a functionally relevant driver of lysosomal or autophagic adaptation that indirectly supports metabolic fitness. The structural proteins FLNC, DSP, and PLEC may reflect broader changes in cellular architecture or state accompanying the drug-resistant phenotype. The PPI panel was included only as known interaction context and should not be interpreted as condition-specific interaction rewiring.

Beyond its canonical enzymatic role in uridine phosphorolysis, emerging evidence suggests that UPP1 may have additional, non-canonical functions relevant to cancer biology. In lung adenocarcinoma, Li et al. demonstrated that UPP1^high^ tumor cells, localized at the invasive front, contribute to an immunosuppressive tumor microenvironment by releasing TGF-β1 and other cytokines, upregulating PD-L1 via the PI3K/AKT/mTOR pathway, and spatially associating with FOXP3 + regulatory T cells, exhausted CD8 + T cells, and immunosuppressive macrophages [46]. This raises the possibility that UPP1 overexpression in PCa may influence tumor biology not only through pyrimidine salvage and nucleotide pool regulation, but also through non-canonical pathways including potential immunomodulation. UPP1 has additionally been linked to epithelial-mesenchymal transition (EMT) and migratory phenotypes in other cancer types [48]. While the present study does not evaluate these functional dimensions, the concordant RNA-protein evidence for UPP1 upregulation in drug-resistant PCa cells, together with the nucleotide remodeling signal in human prostate tissue, provides a rationale for investigating both canonical and non-canonical UPP1 functions in future PCa studies.

The classifier analysis also requires cautious interpretation. Candidate gene sets containing UPP1 showed strong within-layer discrimination, especially in the RNA data and in multi-gene candidate sets. However, nested benchmark models also performed strongly, including a protein nested top-20 model with mean AUC of 1.000. Therefore, the data do not justify presenting a UPP1-centric classifier as outperforming benchmark models. The classification results should be viewed as exploratory evidence that the candidate module carries discriminative information in the available profiles.

This study has several limitations. First, the RNA/protein profiles and ST000784 metabolomics samples were not matched, preventing sample-level correlation, causal modeling, or true three-layer integration. Second, biological replicate identifiers for the processed RNA/protein profile matrices were limited, so profile-level classifier performance may overestimate generalizability if profiles from the same biological replicate are not independent. Third, no perturbation experiment was performed to test whether UPP1 directly regulates N-carbamoyl-L-aspartate, GMP, cAMP, UMP, or uracil abundance. Fourth, the functional interpretation of candidate genes is limited to association evidence; distinguishing between causal drivers, compensatory responses, and bystander effects requires experimental manipulation. Fifth, the classifier results were not externally validated in independent patient cohorts. Current tissue-based genomic tests and biomarker tools in PCa have been adopted unevenly in clinical practice, reinforcing the need for rigorous validation before clinical interpretation [45].

In conclusion, this analysis supports a conservative model in which concordant RNA-protein evidence prioritizes UPP1 as a metabolically interpretable candidate, while independent human matched tissue metabolomics supports broader nucleotide remodeling in PCa. Emerging evidence for non-canonical UPP1 functions, including immunomodulation, adds further motivation for dedicated functional studies. Future studies should use matched transcriptomic, proteomic, and metabolomic measurements from the same specimens, combined with UPP1 perturbation experiments, to test whether UPP1 plays a causal role in PCa nucleotide metabolism, immune regulation, and treatment resistance.

## Conclusions

This study identifies UPP1 as a concordantly upregulated RNA-protein candidate within a broader overlap module and supports a UPP1-associated nucleotide remodeling hypothesis using independent human matched tissue metabolomics. ST000784 data support changes in nucleotide-related metabolites, including N-carbamoyl-L-aspartate, GMP, and cAMP, but do not confirm significant uracil accumulation. Within-layer classifier analyses show that UPP1-containing candidate sets carry discriminative information, although benchmark models also perform strongly. These findings should be considered hypothesis-generating and require matched multi-omics validation and functional testing.

## Supporting information

S1 Data Processed analysis tables needed to reproduce the reported results. (ZIP)

## References

[1] Rawla P. Epidemiology of Prostate Cancer. World J Oncol. 2019;10(2):63–89. doi: 10.14740/wjon1191 31068988 PMC6497009

[2] Kelloff GJ, Choyke P, Coffey DS, Prostate Cancer Imaging Working Group. Challenges in clinical prostate cancer: role of imaging. AJR Am J Roentgenol. 2009;192(6):1455–70. doi: 10.2214/AJR.09.2579 19457806 PMC2893141

[3] Egevad L, Granfors T, Karlberg L, Bergh A, Stattin P. Percent Gleason grade 4/5 as prognostic factor in prostate cancer diagnosed at transurethral resection. J Urol. 2002;168(2):509–13. doi: 10.1016/s0022-5347(05)64669-1 12131299

[4] Stark JR, Perner S, Stampfer MJ, Sinnott JA, Finn S, Eisenstein AS, et al. Gleason score and lethal prostate cancer: does 3 + 4 = 4 + 3?. J Clin Oncol. 2009;27(21):3459–64. doi: 10.1200/JCO.2008.20.4669 19433685 PMC2717753

[5] Tomlins SA, Alshalalfa M, Davicioni E, Erho N, Yousefi K, Zhao S, et al. Characterization of 1577 primary prostate cancers reveals novel biological and clinicopathologic insights into molecular subtypes. Eur Urol. 2015;68(4):555–67. doi: 10.1016/j.eururo.2015.04.033 25964175 PMC4562381

[6] Ren S, Wei G-H, Liu D, Wang L, Hou Y, Zhu S, et al. Whole-genome and Transcriptome Sequencing of Prostate Cancer Identify New Genetic Alterations Driving Disease Progression. Eur Urol. 2018;73(3):322–39. doi: 10.1016/j.eururo.2017.08.027 28927585

[7] Meng J, Lu X, Jin C, Zhou Y, Ge Q, Zhou J, et al. Integrated multi-omics data reveals the molecular subtypes and guides the androgen receptor signalling inhibitor treatment of prostate cancer. Clin Transl Med. 2021;11(12):e655. doi: 10.1002/ctm2.655 34936729 PMC8694501

[8] Bader DA, McGuire SE. Tumour metabolism and its unique properties in prostate adenocarcinoma. Nat Rev Urol. 2020;17(4):214–31. doi: 10.1038/s41585-020-0288-x 32112053

[9] Strmiska V, Michalek P, Eckschlager T, Stiborova M, Adam V, Krizkova S, et al. Prostate cancer-specific hallmarks of amino acids metabolism: Towards a paradigm of precision medicine. Biochim Biophys Acta Rev Cancer. 2019;1871(2):248–58. doi: 10.1016/j.bbcan.2019.01.001 30708041

[10] Zadra G, Photopoulos C, Loda M. The fat side of prostate cancer. Biochim Biophys Acta. 2013;1831(10):1518–32. doi: 10.1016/j.bbalip.2013.03.010 23562839 PMC3766375

[11] Morrish F, Isern N, Sadilek M, Jeffrey M, Hockenbery DM. c-Myc activates multiple metabolic networks to generate substrates for cell-cycle entry. Oncogene. 2009;28(27):2485–91. doi: 10.1038/onc.2009.112 19448666 PMC2779836

[12] Wu X, Daniels G, Lee P, Monaco ME. Lipid metabolism in prostate cancer. Am J Clin Exp Urol. 2014;2(2):111–20. 25374912 PMC4219300

[13] Baron A, Migita T, Tang D, Loda M. Fatty acid synthase: a metabolic oncogene in prostate cancer?. J Cell Biochem. 2004;91(1):47–53. doi: 10.1002/jcb.10708 14689581

[14] Zhang W, Wang T, Wang Y, Zhu F, Shi H, Zhang J, et al. Intratumor heterogeneity and clonal evolution revealed in castration-resistant prostate cancer by longitudinal genomic analysis. Transl Oncol. 2022;16:101311. doi: 10.1016/j.tranon.2021.101311 34902740 PMC8681025

[15] Krycer JR, Brown AJ. Cholesterol accumulation in prostate cancer: a classic observation from a modern perspective. Biochim Biophys Acta. 2013;1835(2):219–29. doi: 10.1016/j.bbcan.2013.01.002 23357067

[16] Masko EM, Allott EH, Freedland SJ. The relationship between nutrition and prostate cancer: is more always better?. Eur Urol. 2013;63(5):810–20. doi: 10.1016/j.eururo.2012.11.012 23219353 PMC3597758

[17] Butler LM, Centenera MM, Swinnen JV. Androgen control of lipid metabolism in prostate cancer: novel insights and future applications. Endocr Relat Cancer. 2016;23(5):R219-27. doi: 10.1530/ERC-15-0556 27130044

[18] Swinnen JV, Esquenet M, Goossens K, Heyns W, Verhoeven G. Androgens stimulate fatty acid synthase in the human prostate cancer cell line LNCaP. Cancer Res. 1997;57(6):1086–90. 9067276

[19] Tousignant KD, Rockstroh A, Taherian Fard A, Lehman ML, Wang C, McPherson SJ, et al. Lipid Uptake Is an Androgen-Enhanced Lipid Supply Pathway Associated with Prostate Cancer Disease Progression and Bone Metastasis. Mol Cancer Res. 2019;17(5):1166–79. doi: 10.1158/1541-7786.MCR-18-1147 30808729

[20] Manzoni C, Kia DA, Vandrovcova J, Hardy J, Wood NW, Lewis PA, et al. Genome, transcriptome and proteome: the rise of omics data and their integration in biomedical sciences. Brief Bioinform. 2018;19(2):286–302. doi: 10.1093/bib/bbw114 27881428 PMC6018996

[21] Pomerantz MM, Li F, Takeda DY, Lenci R, Chonkar A, Chabot M, et al. The androgen receptor cistrome is extensively reprogrammed in human prostate tumorigenesis. Nat Genet. 2015;47(11):1346–51. doi: 10.1038/ng.3419 26457646 PMC4707683

[22] Chen S, Huang V, Xu X, Livingstone J, Soares F, Jeon J, et al. Widespread and Functional RNA Circularization in Localized Prostate Cancer. Cell. 2019;176(4):831-843.e22. doi: 10.1016/j.cell.2019.01.025 30735634

[23] Yang F, Chen Y, Shen T, Guo D, Dakhova O, Ittmann MM, et al. Stromal TGF-β signaling induces AR activation in prostate cancer. Oncotarget. 2014;5(21):10854–69. doi: 10.18632/oncotarget.2536 25333263 PMC4279415

[24] Drake JM, Paull EO, Graham NA, Lee JK, Smith BA, Titz B, et al. Phosphoproteome Integration Reveals Patient-Specific Networks in Prostate Cancer. Cell. 2016;166(4):1041–54. doi: 10.1016/j.cell.2016.07.007 27499020 PMC4985183

[25] Kwon OK, Ha Y-S, Na A-Y, Chun SY, Kwon TG, Lee JN, et al. Identification of Novel Prognosis and Prediction Markers in Advanced Prostate Cancer Tissues Based on Quantitative Proteomics. Cancer Genomics Proteomics. 2020;17(2):195–208. doi: 10.21873/cgp.20180 32108042 PMC7078833

[26] Sun R, A J, Yu H, Wang Y, He M, Tan L, et al. Proteomic landscape profiling of primary prostate cancer reveals a 16-protein panel for prognosis prediction. Cell Rep Med. 2024;5(8):101679. doi: 10.1016/j.xcrm.2024.101679 39168102 PMC11384950

[27] Lee BH, Taylor MG, Robinet P, Smith JD, Schweitzer J, Sehayek E, et al. Dysregulation of cholesterol homeostasis in human prostate cancer through loss of ABCA1. Cancer Res. 2013;73(3):1211–8. doi: 10.1158/0008-5472.CAN-12-3128 23233737 PMC3563867

[28] Sreekumar A, Poisson LM, Rajendiran TM, Khan AP, Cao Q, Yu J, et al. Metabolomic profiles delineate potential role for sarcosine in prostate cancer progression. Nature. 2009;457(7231):910–4. doi: 10.1038/nature07762 19212411 PMC2724746

[29] Kaushik AK, Vareed SK, Basu S, Putluri V, Putluri N, Panzitt K, et al. Metabolomic profiling identifies biochemical pathways associated with castration-resistant prostate cancer. J Proteome Res. 2014;13(2):1088–100. doi: 10.1021/pr401106h 24359151 PMC3975657

[30] Hasin Y, Seldin M, Lusis A. Multi-omics approaches to disease. Genome Biol. 2017;18(1):83. doi: 10.1186/s13059-017-1215-1 28476144 PMC5418815

[31] Menyhárt O, Győrffy B. Multi-omics approaches in cancer research with applications in tumor subtyping, prognosis, and diagnosis. Comput Struct Biotechnol J. 2021;19:949–60. doi: 10.1016/j.csbj.2021.01.009 33613862 PMC7868685

[32] Mashima T, Seimiya H, Tsuruo T. De novo fatty-acid synthesis and related pathways as molecular targets for cancer therapy. Br J Cancer. 2009;100(9):1369–72. doi: 10.1038/sj.bjc.6605007 19352381 PMC2694429

[33] Zhao SG, Chen WS, Li H, Foye A, Zhang M, Sjöström M, et al. The DNA methylation landscape of advanced prostate cancer. Nat Genet. 2020;52(8):778–89. doi: 10.1038/s41588-020-0648-8 32661416 PMC7454228

[34] Vahabi N, Michailidis G. Unsupervised Multi-Omics Data Integration Methods: A Comprehensive Review. Front Genet. 2022;13:854752. doi: 10.3389/fgene.2022.854752 35391796 PMC8981526

[35] Atanassov BS, Mohan RD, Lan X, Kuang X, Lu Y, Lin K, et al. ATXN7L3 and ENY2 Coordinate Activity of Multiple H2B Deubiquitinases Important for Cellular Proliferation and Tumor Growth. Mol Cell. 2016;62(4):558–71. doi: 10.1016/j.molcel.2016.03.030 27132940 PMC4874879

[36] Rodriguez H, Zenklusen JC, Staudt LM, Doroshow JH, Lowy DR. The next horizon in precision oncology: Proteogenomics to inform cancer diagnosis and treatment. Cell. 2021;184(7):1661–70. doi: 10.1016/j.cell.2021.02.055 33798439 PMC8459793

[37] Zhao SG, Chang SL, Erho N, Yu M, Lehrer J, Alshalalfa M, et al. Associations of Luminal and Basal Subtyping of Prostate Cancer With Prognosis and Response to Androgen Deprivation Therapy. JAMA Oncol. 2017;3(12):1663–72. doi: 10.1001/jamaoncol.2017.0751 28494073 PMC5824281

[38] Stelloo S, Nevedomskaya E, Kim Y, Schuurman K, Valle-Encinas E, Lobo J, et al. Integrative epigenetic taxonomy of primary prostate cancer. Nat Commun. 2018;9(1):4900. doi: 10.1038/s41467-018-07270-2 30464211 PMC6249266

[39] Gómez-Cebrián N, Poveda JL, Pineda-Lucena A, Puchades-Carrasco L. Metabolic Phenotyping in Prostate Cancer Using Multi-Omics Approaches. Cancers (Basel). 2022;14(3):596. doi: 10.3390/cancers14030596 35158864 PMC8833769

[40] Woo J, et al. Single-cell proteomic characterization of drug-resistant prostate cancer cells reveals molecular signatures associated with morphological changes. Molecular & Cellular Proteomics. 2025;24(4):100949. 10.1016/j.mcpro.2025.100949 PMC12008537 40090465

[41] Deutsch EW, Bandeira N, Perez-Riverol Y, Sharma V, Carver JJ, Mendoza L, et al. The ProteomeXchange consortium at 10 years: 2023 update. Nucleic Acids Res. 2023;51(D1):D1539–48. doi: 10.1093/nar/gkac1040 36370099 PMC9825490

[42] Metabolomics Workbench. 2017. doi: 10.21228/M80D5X

[43] Szklarczyk D, Kirsch R, Koutrouli M, Nastou K, Mehryary F, Hachilif R, et al. The STRING database in 2023: protein-protein association networks and functional enrichment analyses for any sequenced genome of interest. Nucleic Acids Res. 2023;51(D1):D638–46. doi: 10.1093/nar/gkac1000 36370105 PMC9825434

[44] Pedregosa F, et al. Scikit-learn: machine learning in Python. Journal of Machine Learning Research. 2011;12:2825–30.

[45] Bologna E, Ditonno F, Licari LC, Franco A, Manfredi C, Mossack S. Tissue-Based Genomic Testing in Prostate Cancer: 10-Year Analysis of National Trends on the Use of Prolaris, Decipher, ProMark, and Oncotype DX. Clinics and Practice. 2024;14(2):508–20. doi: 10.3390/clinpract14020039 38525718 PMC10961791

[46] Li Y, Jiang M, Aye L, Luo L, Zhang Y, Xu F, et al. UPP1 promotes lung adenocarcinoma progression through the induction of an immunosuppressive microenvironment. Nat Commun. 2024;15(1):1200. doi: 10.1038/s41467-024-45340-w 38331898 PMC10853547

[47] Cao D, Russell RL, Zhang D, Leffert JJ, Pizzorno G. Uridine phosphorylase (-/-) murine embryonic stem cells clarify the key role of this enzyme in the regulation of the pyrimidine salvage pathway and in the activation of fluoropyrimidines. Cancer Res. 2002;62(8):2313–7. 11956089

[48] Wang J, Xu S, Lv W, Shi F, Mei S, Shan A, et al. Uridine phosphorylase 1 is a novel immune-related target and predicts poor prognosis and immunotherapeutic response in multiple cancer types. Scientific Reports. 2022;12:17392.36253408

[49] Ghosh P, Dahms NM, Kornfeld S. Mannose 6-phosphate receptors: new twists in the tale. Nat Rev Mol Cell Biol. 2003;4(3):202–12. doi: 10.1038/nrm1050 12612639

[50] Martin-Kleiner I, Gall Troselj K. Mannose-6-phosphate/insulin-like growth factor 2 receptor (M6P/IGF2R) in carcinogenesis. Cancer Lett. 2010;289(1):11–22. doi: 10.1016/j.canlet.2009.06.036 19646808

